# Management of ‘forgotten’ encrusted JJ stents using extracorporeal shockwave lithotripsy: A single-centre experience

**DOI:** 10.1080/2090598X.2019.1595485

**Published:** 2019-05-15

**Authors:** Hasan El-Tatawy, Ahmed S. El-Abd, Tarek A. Gameel, Ahmed R. Ramadan, Mohamed O. Abo Farha, Magdy A. Sabaa, Shawky A. El-Abd

**Affiliations:** Department of Urology, Faculty of Medicine, Tanta University, Tanta, Egypt

**Keywords:** Encrustation, JJ stent, ESWL, endourology

## Abstract

**Objective**: To evaluate the role of extracorporeal shockwave lithotripsy (ESWL) for the management of ‘forgotten’ encrusted stents.

**Patients and Method**: This is a retrospective study of 133 patients with forgotten JJ stents, treated between January 2015 and January 2018. Encrustation was mainly found in the renal coil of the stent with distal concomitant encrustation in the vesical and/or ureteric segment. After laboratory and radiological assessment, treatment started with ESWL for the renal encrustation before successful extraction. Auxiliary endourological procedures were used for the encrusted vesical or ureteric segments. Failed cases underwent open surgery.

**Results**: The mean (SD; range) JJ stent indwelling time was 25.84 (10; 14–70) months. In all, 96 (72.2%) patients were seen after treatment for stone disease. In total, 94 patients (70.7%) were managed by ESWL monotherapy, whilst in 36 (27%) additional endourological procedures were required before successful extraction including: cystolithotripsy 19 patients (52.8%), ureteroscopic lithotripsy eight (22.2%), and percutaneous nephrolithotomy nine (25%). Open surgery was required in only three patients (2.3%). A mean of 0.28 procedures per patient was required before smooth stent extraction. The encrusted stents were removed after the first, second, third, and fourth ESWL sessions in 44 patients (33.1%), 43 (32.3%), 26 (19.5%), and 17 (12.8%), respectively. Patients with forgotten indwelling JJ stents for >2 years had significantly larger and harder encrustation at both JJ coils.

**Conclusion**: ESWL proved a feasible first-line treatment for forgotten encrusted JJ stents. The indwelling time of forgotten stents in the urinary tract is associated with greater encrustation burden, density and multiple sites of encrustation.

**Abbreviations:** CLT: cystolithotripsy; ESWL: extracorporeal shockwave lithotripsy; HU: Hounsfield unit; KUB: plain abdominal radiograph of the kidneys, ureters and bladder; PCNL: percutaneous nephrolithotomy; URL: ureteroscopic lithotripsy

## Introduction

Stenting the ureter with a JJ stent is an essential part of many urological procedures, whether needed following open or endoscopic ureteric surgery [1]. Sometimes it can be placed preoperatively to avoid iatrogenic intraoperative ureteric injury during major pelvic operations for gynaecological and colorectal surgery. It has also been used as a lifesaving procedure in cases with calcular anuria and ureteric obstruction with infection.

Urologists have experienced an increased incidence of encrusted stents neglected by uncompliant patients. The ‘forgotten’ stent may be asymptomatic and only an abdominal imaging incidentally reveals its presence. If the stent is forgotten for a long time, possible complications may occur such as urinary tract infection (UTI), stone formation, fragmentation, migration; or encrustation and more seriously secondary renal dysfunction. Whilst, patients with ureteric obstruction from an encrusted forgotten stent can present with life-threatening urosepsis, which may be lethal in some cases [2–4]. Many factors promote the process of encrustation including the material of the stent, urine composition, and duration of contact of the stent with urine [5]. The exact mechanism of encrustation is unclear but it seems to be dependent on pH, ionic strength, and biomaterial hydrophobic properties. The biochemical and optical analysis of stent encrustation has shown that encrustations consist mainly of calcium oxalate, calcium phosphate, and ammonium magnesium phosphate [6,7].

The management of forgotten JJ stent constitutes a dilemma to the urologist and sometimes may be difficult, complicated, risky and expensive [8]. Although open surgery has been reported as a treatment modality, other minimally invasive procedures are followed; of these techniques, extracorporeal shockwave lithotripsy (ESWL), or internal lithotripsy with percutaneous nephrolithotomy (PCNL), cystolithotripsy (CLT), ureteroscopic lithotripsy (URL) have all been used either alone or in combination to tackle this problem [8,9].

In our present study, we present the results of a single-centre study of ESWL as an initial management of patients with forgotten encrusted JJ stents.

## Patients and methods

This is a retrospective study of 133 patients with forgotten ureteric stents treated in the Department of Urology, Tanta University, Egypt, between January 2015 and January 2018. In all, 103 patients were referred from other hospitals and centres. All patients had forgotten encrusted JJ stents that had been left *in situ* for a long time and managed initially by ESWL. The presenting symptoms were an association of symptoms in the form of attacks of fever in 100 patients, UTI in 92, recurrent attacks of renal pain and colic in 52, haematuria in 49, obstructed hydronephrosis in 20, and infected non-functioning hydronephrotic kidney in one. All the patients also had LUTS. The preoperative evaluation consisted of: urine analysis with antibiotic sensitivity test, serum creatinine level, complete blood count with coagulation profile, and plain X-ray of the abdomen and pelvis and non-contrast spiral CT to evaluate stone burden density and the sites of stent encrustation.

The first treatment approach for these patients was ESWL for the encrusted stent in the renal pelvis. The machine used was the Dornier Compact Delta® II (Dornier MedTech, Munich, Germany). Patients were placed supine and the encrusted stent was identified by the C-Arm with the head therapy either anterior or posterior according to the perfect adjustment of the target encrustation. The shockwave power started at 8 kV and was incrementally increased to 13 kV, at a rate of 60 shocks/min, and gradually raised to 90 shocks/min according to the recommended table of the machine manufacturer. Radiological monitoring of the encrustation was done until complete disintegration was seen after one–four sessions before considering the ESWL procedure as a failed initial treatment. Failed cases underwent other endourological procedures such as PCNL, URL or CLT, whilst open surgery was provided for failed combined treatment or complicated cases only.

The procedures were performed under complete antibiotic cover. Patients with concomitant distal or encrustation along the ureteric segment subsequently underwent CLT with or without URL. After freeing the proximal coil in the renal pelvis, as well the distal or ureteric segment from encrustation, extraction of the JJ stent was done and this was considered as success. Successful patients were followed-up with plain abdominal radiograph of the kidneys, ureters and bladder (KUB) and non-contrast spiral CT after 3–4 weeks.

Analysis of ESWL treatment data and results were correlated to the number of ESWL sessions, indwelling duration of the forgotten JJ stent, radiological density, and size of encrustation. The IBM Statistical Package for the Social Sciences (SPSS®), version 25 (SPSS Inc., IBM Corp., Armonk, NY, USA) was used for analysis. The statistical methods used were descriptive statistics, frequency analysis, Pearson correlation and unpaired *t*-test. Results are expressed as the number of patients (*n*), mean, standard deviation (SD) and range (minimum–maximum). A *P* < 0.05 was considered to be statistically significant.

## Results

Amongst 133 patients, 70.7% (*n* = 94) were males and 29.3% (*n* = 39) were females, with a mean (SD; range) age of 44.15 (8; 27–63) years. The mean (SD; range) indwelling time of the JJ stent was 25.84 (10; 14–70) months.

Out of 103 patients referred from other centres, 16 patients (15.5%) were unaware of the JJ placement. The encrustation was along the whole length of the stent in three patients (2.26%), whilst in 130 patients the encrustation was in the upper coil and also in the ureter and lower coil in 22 and 19 patients, respectively. Urine culture was positive in 52 patients (39%) treated prior to intervention.

The preoperative site and burden of encrustation evaluated by KUB and non-enhanced spiral CT was important to decide the treatment plane after initial ESWL. The reasons for JJ placement were identified from the reports as: post-pyeloplasty (16 patients), endoscopic dilatation of the ureter (nine), obstructive uropathy during pregnancy (six), open ureterolithotomy (20), post uretero-vesical re-implantation (five), and in the majority of patients (77) the stent was inserted for ureterorenoscopy and stone extraction. Patients with the encrusted stent in the upper coil only and of <10 mm, the mean encrustation size was 9.13 mm, whilst it was 17.3 mm in 26 patients with encrustation size of 10–20 mm, and 22 mm in one patient with encrustation burden of >20 mm.

In patients with encrusted JJ stents either in both upper and lower coil, and both upper coil and ureter, the mean encrustation size was almost equal (15.5 and 15.9 mm; Table 1).

**Table 1.** The site and size of encrustions in relation to indwelling time of forgotten JJ stents in 130 patients.

Indwelling time		Upper coil (<10 mm)	Upper coil (10–20 mm)	Upper coil (>20 mm)	Upper coil and ureter (mm)	Upper and lower coil (mm)	HU
≤24 month	*N*	52	26	1			79
	Mean (SD)	9.13 (1.1)	17.3 (1.9)	22.0			707.6 (113.8)
	Range	6–10	15–20	22–22			500–950
>24 month	*N*			10	22	19	51
	Mean (SD)			23.9 (1.6)	15.9 (8.7)	15.5 (3.3)	863.9 (187.4)
	Range			22–26	7–30	10–20	500–1300
*P*				0.285			<0.001

**Figure 1.**
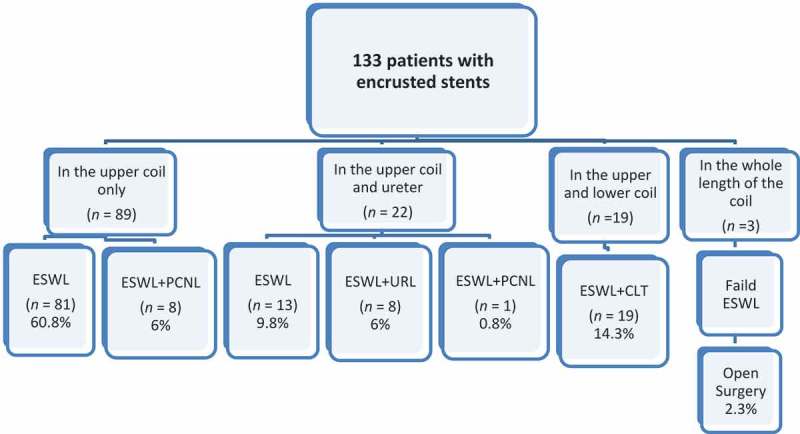
Treatment data according to sites of encrustation.

After ESWL treatment the encrusted stent was easily removed with the cystoscope in 94 patients (70.7%). In a further 36 patients (27%) a combination of ESWL with other endourological procedures was needed; 19 patients had CLT, nine had PCNL, and eight had URL. Only three patients (2.3%) with an encrusted entire length of the stent failed combined endourological procedure with ESWL after four sessions and required open surgery (Figure 1).

Of the 94 patients successfully treated by ESWL monotherapy; 41 patients (43.6%) required one session, 32 (34%) required two sessions, whilst 13 (13.8%) and eight (8.5%) required three and four sessions, respectively.

Patients with combined ESWL and CLT (*n* = 19), underwent endoscopy after their first, second and third ESWL session in three patients (15.8%), 11 (57.9%) and five (26.3%), respectively, for patients having encrusted upper and lower coils. In nine cases initial ESWL failed to clear the encrustation but PCNL was done after the third and fourth sessions in eight patients and one, respectively, whilst URL was needed in eight patients after the fourth ESWL sessions before smooth endoscopic extraction of the stent. In five patients, due to intense ureteric manipulation re-insertion of a JJ stent was necessary for 2 weeks before safe removal. With or without auxiliary procedures to tackle the encrustation the stent was removed after the first, second, third and fourth ESWL sessions in 44 patients (33.9%), 43 (33%), 26 (20%) and 17 (13%), respectively (Table 2).

**Table 2.** Role of ESWL and endourolgical procedures in successful treatment (130 patients).

No. of ESWL sessions		ESWL + endourological auxiliary procedure			Total*
	ESWL monotherapy	ESWL + CLT	ESWL + URL	ESWL + PCNL	
	*N*	*N*	*N*	*N*	*N* (%)
One	41	3	–	–	44 (33.9)
Two	32	11	–	–	43 (33)
Three	13	5	–	8	26 (20)
Four	8	–	8	1	17 (13.1)
Total, n/N (%)	94	19/36	8/36	9/36	130/133 (97.7)
	94/133(70.7)	36/133 (27)			

*Three cases failed.

In 79 patients with forgotten stents left *in situ* for <2 years, there was only encrustation of the upper coil compared to extra-renal distal encrustation of larger size with more dense and hard encrustation in 51 patients with stents left *in situ* for >2 years.

**Figure 2.**
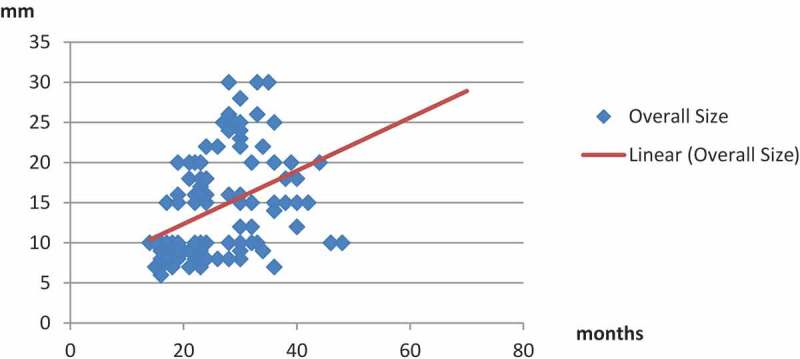
Correlation between stent indwelling time and overall encrustation (stone) size.

Overall, the mean (SD; range) duration of indwelling JJ stent was 25.8 (9.5; 14–70) months and the mean (SD; range) size of encrustation was 14 (6.1; 6–30) mm, with the size of encrustation increasing significantly with time (*P* < 0.001, with a moderate positive Pearson Correlation *r* = 0.425; Figure 2).

The mean (SD; range) density of encrustation was 707.6 (113.8; 500–950) Hounsfield units (HU) in patients with forgotten stents for <2 years compared to 863.6 (187.4; 500–1300) HU in patients with longer durations of forgotten stents, and the difference was statistically significant (*P* < 0.001).

**Figure 3.**
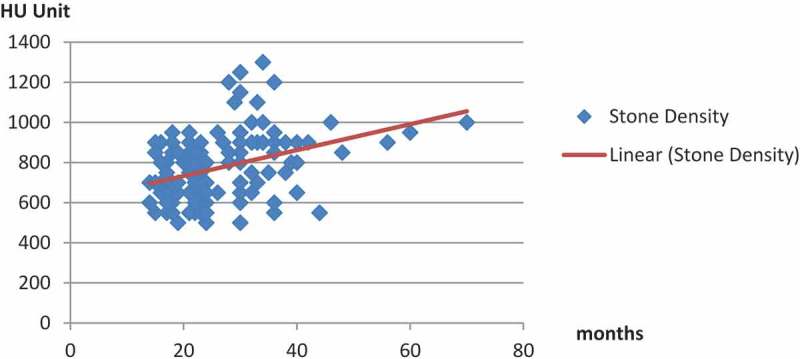
Correlation between stent indwelling time and encrustation (stone) density.

A longer duration of forgotten stent was significantly moderately correlated with harder encrustation of the stent and the subsequent need for a more complex treatment regimen (Table 1), with a positive Pearson correlation *r* = 0.386 (*P* < 0.001; Figure 3).

In all patients, after complete freeing of the stent from encrustations after ESWL monotherapy and in cases who underwent combined endourological treatment, the extraction of the stent was done under continuous fluoroscopic monitoring to ensure a loose proximal stent (upper coil). The stent removal rate after ESWL, with and without endourological procedures, was 97.7%. A total of 36 endourological procedures had to be performed before safe extraction with a mean of 0.28. Complications encountered during or after treatment were all minor and managed conservatively without surgical intervention. These complications comprised fever (*n* = 16), septicaemia (*n* = 5), and minute ureteric extravasation in two patients, all managed with i.v. fluids and antibiotics according to the preoperative antibiogram, with transient ureteric stenting for 3 and 5 days.

## Discussion

Ureteric stents have been used widely in urology since their first introduction in 1967 [10]. They are mainly used for preventing or managing obstruction within the urinary tract secondary to variety of causes; calcular disease, pregnancy, malignancy, and oedema after reconstructive surgeries. In the present study, 72.2% of the forgotten stents were seen following open and endourological treatment of stones disease, as has been previously observed by other authors [11].

A JJ stent, when indicated for a long period must be changed after 3–4 months to avoid complications. However, along with its positive contributions, the stent may cause complications such as pain, haematuria, dysuria, irritative symptoms, and fever in the early period after placement (3–9 weeks). The most common complications in the long-term are UTI, migration, encrustation, fragmentation, blockage, and hydronephrosis [12]. In our case series with forgotten JJ stents, the most prominent complication was LUTS followed by fever and UTI.

The encrusted stent has many terms throughout literature; the ‘retained stent’, ‘neglected stent’, ‘forgotten stent’, and ‘overlooked stent’. It may be asymptomatic and found only when it appears fortuitously by abdominal imaging, as was the case in 15.5% in our present series. Conversely, a patient with ureteric obstruction from an encrusted stent can present with life-threatening urosepsis, which may be lethal in some cases [13]. The problem of encrustation of indwelling urinary stents can be attributed to many factors. The chemical constituents of urine combined with the tubing can form a matrix, on which further calcification occurs; with an end result of encrustation. Various factors contribute to the rate at which the process occurs, including the stent material, urine composition and pH, and duration of urine contact with the stent [2].

In the presence of encrustation, every endoscopic manipulation of forgotten stents should first and always be preceded by appropriate imaging to decide the safest removal strategy. Second, force should be avoided if removal of the stent cannot be managed by a simple cystoscope [14]. Combined endourological intervention, or rarely an open surgical approach may be needed for their management.

In our present cases, 97.7% of the neglected stents were extracted cystoscopically with no need for open intervention, of which 70.7% were successfully treated by ESWL monotherapy. A combination of ESWL and an endourological procedure was mandatory prior to smooth extraction of the stent in 27% of patients. Only three of our present patients (2.3%) required open surgery, as the stent could not be removed after four sessions of ESWL due to the longevity of stent indwelling (~5 years) and its location (the whole length of the stent). Anwar et al. [15] showed that 87.5% of their study cases were managed endoscopically with a 43.8% success rate with a single cystoscopic procedure, whilst combined ESWL with endoscopic procedures was required in rest of the cases (43.6%). The open procedure was required in two cases following failed attempted URL.

Using a combination of ESWL, PCNL, CLT, and URL, clearance rates ranging from 75% to 100% have been reported [16]. In our present study, we have found that in 19/36 patients (52.8%) with encrustation of the stent in the lower coil, the stent could not be removed with ESWL monotherapy, but was easily removed with the aid of CLT after one or more sessions of ESWL. Also, in eight patients (22.2%) with encrustation in the upper coil and ureter, URL was required after four sessions of ESWL, whilst nine (25%) patients required PCNL after the third or fourth session of unsuccessful ESWL.

The duration that the stent remains in the urinary tract is associated with more encrustation burden, density and multiple sites of precipitation. El-Faqih et al. [17] stated that the stent encrustation rate rises from 9.2% before 6 weeks to 47.5% between 6 and 12 weeks, and up to 76.3% after 12 weeks. In our present series overall, the mean (SD; range) indwelling time was 25.84 (9.5; 14–70) months and the mean (SD; range) size of encrustation was 14 (6.1; 6–30) mm, and the size of this encrustation increased significantly with time (*P* < 0.001, with a moderate positive Pearson correlation *r* = 0.425).

Singh et al. [18] in 2001, recommended a classification for management depending on the size and site of encrustation, where slight encrustation (<1 cm), can be removed by cystoscopy after two sessions of ESWL. The same study determined that for intense and large encrustation (>4 cm), ESWL alone may not be appropriate, and it was recommended that combined endourological approaches, such as URL or PCNL, should be used to enable removal of the stent. Also, Irkilata et al. [14] reported that encrustation was generally slight (<1 cm) in their study and one session was found to be sufficient for removal of the stent in 21 of 44 patients. They also reported that two sessions of ESWL were required for eight of their patients and only one patient required three sessions of ESWL. In our present study, the mean (SD) size of encrustation was found to be 9.13 (1.1) mm for patients with slight encrustation (<1 cm) in the upper coil, whilst it was 23.9 (1.6) mm for patients with encrustations of >2 cm. Contrary to both aforementioned studies, we found that one session was sufficient for removal of the stent in 41 of 52 patients having encrustations of <1 cm, whilst two sessions of ESWL were required for 11 of the patients. For the upper coil encrustation of >2 cm, ESWL alone may not be appropriate, and it is recommended that endourological approaches, such as PCNL, be used to enable removal of the JJ stent after ESWL.

Recent studies have shown that HU values affect ESWL results (number of shocks, session, and success) [19]. In our present study, the mean (SD) density of encrustation was 707.6 (113.8) HU in patients with forgotten stents left *in situ* for <2 years, compared to a mean (SD) of 863.6 (187.4) HU in patients with forgotten stents left *in situ* for longer (*P* < 0.001). The overall indwelling time and stone density were moderately positive correlated (*r* = 0.386). The results of our present study concur with the Tarawneh et al. [20] observation that stones with a density of ≤950 HU undergo successful ESWL monotherapy treatment with a lesser number of sessions. Therefore, ESWL treatment outcome is inversely dependent on indwelling time, stone density, and stone size.

In our present study, the stent removal rate after ESWL, with and without endourological procedures, was 97.7%. The mean number of endourological procedures was 0.28, compared to a mean of 1.9 procedures/patient for stent removal by other authors [8]. Complications encountered during or after treatment were all minor and managed conservatively without surgical intervention.

Inadequate communication between the surgeon and patient, and poor compliance are the main factors associated with JJ stent retention. Patients should be counselled properly and made aware of the importance of stent presence, complications, and removal. Also different methods can be followed depending on recent telecommunication tools, e.g. telephone calls, SMS (short message service) texts and e-mails, to avoid this possible problem [21]. Furthermore between stent placement and removal, patients should be advised on how to reduce encrustations, such as adequate hydration, and the use of inhibitors of crystals growth and aggregation [2].

Limitations of the present study include the lack of standardisation of stent material, and the lack of information about the composition of the stents, in addition to the lack of data about preoperative renal function, and the situation at the time of stent placement. That is because 77.5% of our cases were referred from other centres.

## Conclusion

In the present study, forgotten encrusted JJ stents were safely treated with ESWL alone in more than two-thirds of patients and in one-third with auxiliary endourological procedures. Open surgery is rarely needed. The duration that the stent remains in the urinary tract is associated with more encrustation burden, density and multiple sites of precipitation, which reflects on the success of ESWL, the number of sessions required, and the need for a more complex treatment protocol.
